# Optimization of linkage mapping strategy and construction of a high-density American lotus linkage map

**DOI:** 10.1186/1471-2164-15-372

**Published:** 2014-05-15

**Authors:** Qiong Zhang, Leiting Li, Robert VanBuren, Yanling Liu, Mei Yang, Liming Xu, John E Bowers, Caihong Zhong, Yuepeng Han, Shaohua Li, Ray Ming

**Affiliations:** Key Laborary of Plant Germplasm Enhancement and Specialty Agriculture, Wuhan Botanical Garden, Chinese Academy of Sciences, Wuhan, 430074 P.R. China; Department of Plant Biology, University of Illinois at Urbana-Champaign, Urbana, Illinois 61801 USA; College of Horticulture, Nanjing Agricultural University, Nanjing, 210095 P.R. China; Department of Crop and Soil Sciences, University of Georgia, Athens, GA 30602 USA; FAFU and UIUC-SIB Joint Center for Genomics and Biotechnology, Fujian Agriculture and Forestry University, Fuzhou, Fijian 350002 China

**Keywords:** Chinese lotus, Genome assembly, Genotyping by sequencing, Restriction associated sequencing, Megascaffold

## Abstract

**Background:**

Lotus is a diploid plant with agricultural, medicinal, and ecological significance. Genetic linkage maps are fundamental resources for genome and genetic study, and also provide molecular markers for breeding in agriculturally important species. Genotyping by sequencing revolutionized genetic mapping, the restriction-site associated DNA sequencing (RADseq) allowed rapid discovery of thousands of SNPs markers, and a crucial aspect of the sequence based mapping strategy is the reference sequences used for marker identification.

**Results:**

We assessed the effectiveness of linkage mapping using three types of references for scoring markers: the unmasked genome, repeat masked genome, and gene models. Overall, the repeat masked genome produced the optimal genetic maps. A high-density genetic map of American lotus was constructed using an F_1_ population derived from a cross between *Nelumbo nucifera* ‘China Antique’ and *N. lutea* ‘AL1’. A total of 4,098 RADseq markers were used to construct the American lotus ‘AL1’ genetic map, and 147 markers were used to construct the Chinese lotus ‘China Antique’ genetic map. The American lotus map has 9 linkage groups, and spans 494.3 cM, with an average distance of 0.7 cM between adjacent markers. The American lotus map was used to anchor scaffold sequences in the *N. nucifera* ‘China Antique’ draft genome. 3,603 RADseq markers anchored 234 individual scaffold sequences into 9 megascaffolds spanning 67% of the 804 Mb draft genome.

**Conclusions:**

Among the unmasked genome, repeat masked genome and gene models, the optimal reference sequences to call RADseq markers for map construction is repeat masked genome. This high density genetic map is a valuable resource for genomic research and crop improvement in lotus.

## Background

The Nelumbonaceae is a perennial, aquatic plant family, which comprises only one genus, *Nelumbo,* consisting of two species: Chinese lotus *N. nucifera* Gaertn (Asia, Australia, Russia) and American lotus *N. lutea* Pers (eastern and southern North America) [1]. Lotus is a diploid plant (2n = 2× = 16) with agricultural, medicinal, and ecological significance. Chinese lotus (*N. nucifera* Gaertn) is cultivated for its edible rhizomes, seeds, and leaves, which have been consumed as food for thousands of years in Asia. Nearly all parts of lotus have been used as herbal medicines to treat cancer, depression, diarrhea, heart problems, hypertension, insomnia, pyrexia, and obesity [2–4]. Lotus has been shown to be an effective phytoremediator, playing a critical role in removal of heavy metals from polluted water [5, 6]. *N. nucifera* ‘Chinese Antique’ and *N. lutea* ‘AL1’ are geographically isolated and have distinct morphological traits [7]. American lotus has been used to transfer desirable ornamental flower traits to Chinese lotus germplasm.

Despite the agricultural and medicinal importance of lotus, only limited genetic resources are available for genome analysis and molecular breeding. In recent years, a range of DNA markers have been developed for assessment of genetic diversity and population structure in lotus, including simple sequence repeats (SSRs) [8, 9], random amplified polymorphic DNA (RAPD) [10], inter-simple sequence repeats (ISSRs) [11], sequence-related amplified polymorphisms (SRAPs) [7] and amplified fragment length polymorphisms (AFLPs) [12]. These resources are inadequate for comparative genomics and analysis of quantitative trait loci (QTL). Genetic linkage maps are an essential resource for studies on genome structure, genome evolution, and for identification of Mendelian components of QTLs [13].

Two genetic maps was constructed based on SSR markers using an F_1_ population derived from a cross between ‘Chinese lotus’ and ‘American lotus’ [7]. However, the resulting genetic maps of these two parents consist of 47 and 177 markers, respectively. These are the first genetic maps of lotus, and the sequence based SSRs make them a good resource for genome assembly. However, for a genome sequencing project, a much higher density genetic map with sequence-tagged markers is necessary to anchor scaffolds on linkage groups or chromosomes.

Single nucleotide polymorphism (SNP) markers have become the marker system of choice because of the high abundance of source polymorphisms in genome sequences and the automation of allele calling [14]. To generate high-density genetic maps, automated high-throughput, low-cost new technologies for molecular marker genotyping are required. Recently, a significant advance in genotyping using restriction-site associated DNA sequencing (RADseq), has allowed rapid discovery of thousands of SNPs and InDel markers, and high throughput genotyping of large populations [15, 16]. Restriction-site associated DNA sequencing has been effectively applied in genetic mapping and QTLs analyses in eggplant [17], barley [18], *Cynara cardunculus*[19], *Lolium perenne*[20], grape [21] and other plants.

RADseq markers are high throughput and cost effective for genetic mapping. However, scoring RADseq markers presents a computational challenge, and a range of reference sequences can be used. One of our objectives is to identify optimal reference sequences for scoring RADseq markers. In the absence of a draft genome assembly, the most common references currently are either parental reads assembled to form clusters or gene models. Parental RADseq tags have been assembled into clusters and used as references for assigning offspring genotypes in plants [17, 19] and animals [22, 23]. Alternatively, gene models from a closely related species can be used as references for scoring RADseq markers in barley [18]. Both of these approaches have shortcomings which limit the number of callable markers. Homologous clusters can be repetitive and lead to erroneous marker calling and gene models can exclude single copy non-coding regions. A draft genome sequence provides the best reference [24, 25], but still produces a range of various quality markers depending on the input sequence. Few research on employing repeat masked genome as reference in RADseq marker discovery was reported.

Here, we report the construction of two high-resolution genetic linkage maps for lotus using RADseq markers and a segregating F_1_ population crossed between *Nelumbo nucifera* ‘Chinese Antique’ and *N. lutea* ‘AL1’, the same mapping population used for linkage mapping with SSR markers [7]. We tested multiple strategies for scoring RADseq markers and selected the optimal one for constructing a high density genetic map of ‘AL1’ and a low density map of ‘Chinese Antique’ due to extremely low heterozygosity within the genome. The ‘AL1’ genetic map was used for genome assembly of the lotus genome and anchored 68% of the assembled genome [26]. The genetic map will facilitate the identification of genes controlling horticultural, medicinal, and ornamental traits, and provide a reference for large-scale re-sequencing projects.

## Results

### RADseq tag sequencing and marker development

RADseq libraries were sequenced using Illumina HiSeq2000, generating a total of 106 million 100 bp reads. After sorting reads by F_1_ individuals, sequences were trimmed to 92 bp to remove the 4–8 bp Illumina barcode sequences. Low quality reads (reads appearing five nucleotides with Q score < 20), and ambiguous reads with incorrect barcodes were excluded, leaving 97.6 million reads for further analysis. Of these high-quality reads, 8.6 million reads were from female parent *N. nucifera* ‘Chinese Antique’, and 5.9 million reads were from the male parent *N. lutea* ‘AL1’. The number of reads per F_1_ individuals varied from 1.1 million to 4.2 million, with an average read number of 1.9 million per progeny.

Reads were aligned into clusters with the repeat masked megascaffolds as a reference for map construction after comparative analysis of three sets of references (see below), where clusters with less than 4 or more than 200 sequences were discarded to avoid misidentification of polymorphisms due to low coverage or highly repetitive regions respectively. Finally, 26,211 clusters presented more than one genotype in the F_1_ population, with an average coverage of 363.5× for polymorphic loci at the population level. Of the 26,211 polymorphic loci, 22,872 represent homologous loci in ‘China Antique’ and heterozygous loci in ‘AL1’, while 2,777 were homozygous for ‘AL1’ and heterozygous for ‘China Antique’. However, only 562 loci that could be assigned to both maternal and paternal maps were detected, accounting for 2% of all polymorphic loci.

Despite a relative high average coverage for polymorphic RADseq clusters, many RADseq clusters have low coverage in some F_1_ plants. To avoid the errors in data analysis, only tag clusters showing three or more fold coverage of > 90% of all F_1_ individuals were selected. In addition, for controlling marker quality, the missing ratio per potential marker was calculated in the F_1_ population, and the markers with more than 90% integrity were retained. RADseq markers in testcross and intercross configurations are expected to segregate 1:1 and 1:2:1, respectively, markers displaying a segregation distortion (according to the criteria described in “Methods”) were excluded from analysis.

### Optimizing linkage mapping strategy using sequence based markers

An important consideration for calling RADseq markers is the reference sequences used for marker identification. To identify the most effective reference sequences, unmasked scaffolds, the repeat masked scaffolds, and gene models were used as references to call RADseq markers for map construction. The number of scored markers is the highest with unmasked scaffolds, moderate with repeat masked scaffolds, and lowest with gene models at 8,501, 4,098, and 776 markers respectively. However, the percentage of markers anchored to the top 10 megascaffolds (assembled from 234 scaffolds using RADseq markers from the optimal genetic map) is the highest with repeat masked scaffolds, moderate with gene models, and lowest with unmasked scaffolds at 88%, 75%, 65%, respectively, reflecting the accuracy of genetic maps with markers scored using three different references (Table 1).

**Table 1:** **Summary of mapping results from three references**

		Whole genome	Gene models	Masked genome
Megascaffolds	LG	Mapped markers		
**1**	1	1539	131	868
**2**	3	733	89	453
**3**	2	833	96	677
**4**		40	13	5
**5**	8	300	33	208
**6**	5	427	75	434
**7**	4	619	60	337
**8**	6	392	36	232
**9**	7	310	29	229
**10**	9	341	20	160
**Mapped markers**		5534	582	3603
**Scored markers**		8501	776	4098
**% markers anchored**		65	75	88

**Figure 1:**
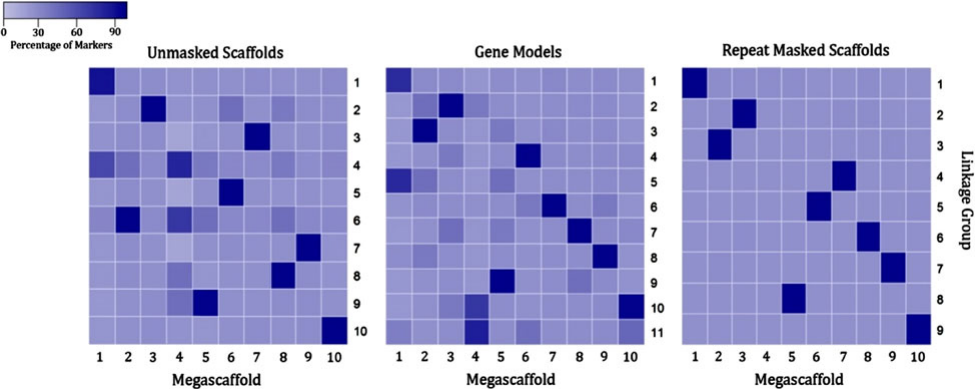
**Distribution of mapped markers using the unmasked**, **masked scaffolds and gene models a reference**. The ten largest scaffolds were used as a reference for calling RADseq markers. The percentage of markers mapping to the linkage groups for each scaffold are shown, with dark blue indicating most or all markers on the scaffold map to one linkage group, and light blue indicating no or few markers map to that linkage group.

The distribution of anchored markers on the scaffolds also provides valuable information for assessing the quality of linkage maps using each of the three sets of references for scoring markers. For the linkage map constructed using the unmasked scaffolds as a reference, each megascaffold anchored markers from multiple linkage groups, and megascaffolds 1 and 2 anchored markers from all 9 linkage groups. Each scaffold anchored vast majority of markers from one linkage group using the repeat masked scaffolds as a reference, except megascaffold 4, and the same pattern was observed for linkage map constructed using unmasked scaffolds as references, except megascaffold 1 (Table 2). On megascaffold 1, for markers derived from the unmasked genome, 818 anchored markers were mapped to linkage group (LG) 1, and 489 and 109 markers mapped to LG4 and LG6, respectively. For markers derived from gene models, 79 anchored markers mapped to LG1 and 45 markers mapped to LG5, and for markers derived from the repeat masked genome, 859 anchored markers mapped to LG1 with less than 10 markers mapped to other LGs. On megascaffold 2, 592 markers mapped to LG 6 using the unmasked genome, and 141 markers mapped to other 8 LGs compared to the repeat masked genome where 433 anchored markers mapped to LG3 and only 20 markers mapped to other 4 LGs. Each of the 9 LGs derived from using the repeat masked genome as a reference anchored to one scaffolds unambiguously, cleaner than the other two linkage maps using unmasked genome and gene models as references (Figure 1). For this reason, we report the linkage maps constructed with markers scored using the repeat masked genome as a reference in the following section.

**Table 2:** **Comparison of linkage mapping results using assembled genome**, **gene models**, **and repeat masked genome as reference for scoring RADseq markers**

Unmasked genome as reference												Gene models as reference											Repeat Masked genome as reference								
Linkage group (number of markers)												Linkage group (number of markers)											Linkage group (number of markers)								
Scaffolds	LG	1	2	3	4	5	6	7	8	9	10	1	2	3	4	5	6	7	8	9	10	11	1	2	3	4	5	6	7	8	9
1	1	818	18	30	489	13	109	14	26	8	14	79	0	0	0	45	2	0	3	0	0	2	859	1	0	3	0	0	3	0	2
2	3	21	17	17	35	12	592	15	15	4	5	2	7	75	0	1	2	0	2	0	0	0	6	8	433	0	0	0	5	0	1
3	2	5	743	7	19	2	29	0	3	8	17	2	90	0	3	0	0	1	0	0	0	0	4	669	0	0	0	4	0	0	0
4		2	2	0	14	0	12	0	3	5	2	0	5	0	0	0	1	0	0	0	4	3	3	1	0	0	1	0	0	0	0
5	8	8	7	1	27	8	40	9	3	195	2	0	0	1	0	0	0	1	0	31	0	0	3	0	0	0	0	0	0	205	0
6	5	0	3	1	17	386	17	2	0	1	0	0	0	1	65	0	7	0	0	0	0	2	1	1	2	0	430	0	0	0	0
7	4	6	2	543	21	1	30	1	3	2	10	2	0	2	1	0	55	0	0	0	0	0	0	2	0	335	0	0	0	0	0
8	6	6	5	8	17	0	32	8	297	12	7	0	1	0	0	0	0	34	0	1	0	0	5	3	1	0	0	222	0	0	1
9	7	0	2	0	3	0	11	289	3	1	1	0	0	0	0	0	0	0	29	0	0	0	1	0	2	1	0	0	225	0	0
10	9	15	7	7	21	1	20	0	0	2	268	1	0	0	3	1	0	2	0	0	11	2	1	0	0	0	2	0	1	0	156

*LG*: linkage group.

Deriving markers from the gene models eliminates errors due to repetitive sequences but is still problematic because of multicopy genes. Multicopy genes are likely responsible for the high number of mismatched markers on megascaffold 1, where 79 markers map correctly on LG1 and 45 markers map on LG5 (Table 1, Figure 1). These errors in megascaffold 1 can also be seen in markers derived from the unmasked genome, 818 markers map correctly to LG1, and 489 and 109 markers map incorrectly to LG4 and LG6 respectively. Using the repeat masked genome as a reference eliminated this problem, as 859 markers map to LG1 with less than 10 markers mapping to the other LGs. A reduction of erroneous markers in the repeat masked markers compared to the unmasked and gene model markers can be seen in the other 9 megascaffolds (Figure 1). Megascaffold 2 for instance has 592 markers correctly mapping to LG 6 using the unmasked genome as a reference, but also has 141 markers in the other LGs. The repeat masked megascaffold 2 has 433 correct markers and only 20 erroneous markers. A set of makers aligned to the repeat masked scaffold produced the optimal genetic maps likely because of reduce erroneous marker detection caused by high copy number repetitive elements.

### Genetic maps

Of the 22,872 potential RADseq markers, 4,098 markers were scored and sorted into 634 recombination bins for constructing the male parental (‘AL1’) genetic map. Comparatively fewer markers were identified in the ‘Chinese Antique’ genetic map, only 147 of the 2,777 potential loci were scored.

**Figure 2:**
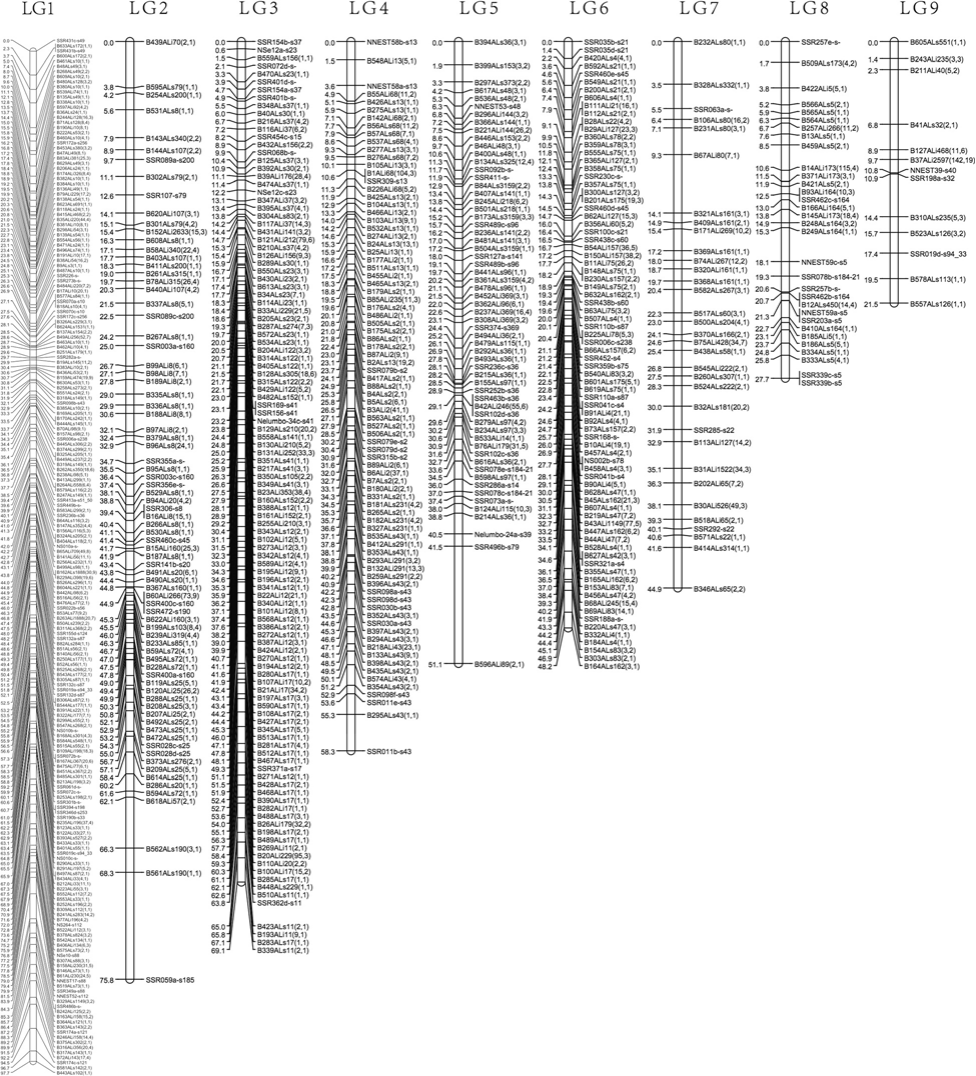
**Paternal linkage maps**.

**Figure 3:**
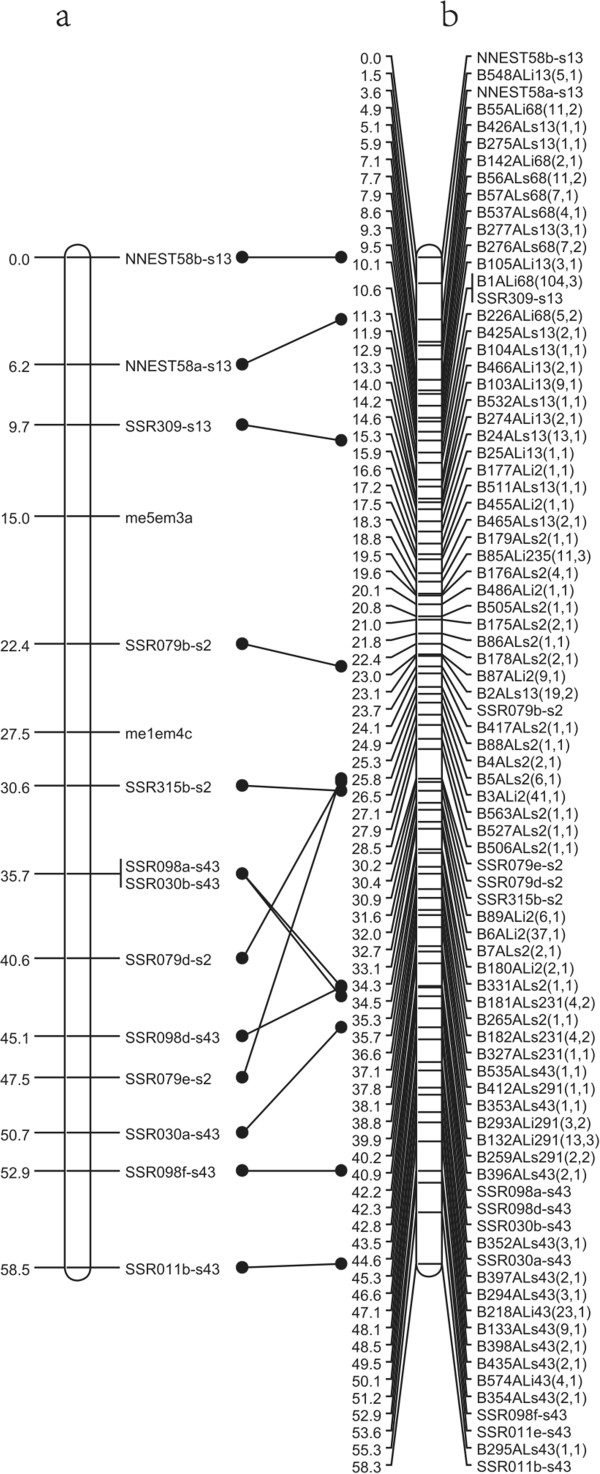
**Comparison of male LG3 in this study with LG5 in previous maps**. The paternal linkage group 3 **(b)** is generated in our study, and the linkage group 5 **(a)** is from previous paternal maps.

A total of 4,234 markers, including 136 lotus SSR markers [7] and 4,098 RADseq markers were used to construct the American lotus genetic map. The 4,098 RADseq markers were assigned to 634 recombination bins, of which 562 bins (3,894 RADseq markers) were integrated with 136 SSR markers to construct the American lotus genetic map. With a LOD threshold of 5.0 and a recombination frequency of 0.25, the regression mapping algorithm in JoinMap 4.1 grouped 562 bins and 136 SSR markers into 9 linkage groups (Figure 2). The number of linkage groups is close to the haploid chromosome number of 8 in lotus, suggesting one chromosome spans into two linkage groups. The total length of the American lotus genetic map is 494.3 cM, with an average distance of 0.7 cM between adjacent markers/bins. Linkage groups have a wide variation in size; the longest linkage group is 97.7 cM, while the shortest is 21.5 cM. The number of markers in each LG varies from 13 in LG9 to 203 in LG1. LG 1 has the highest density of markers with an average interval of 0.48 cM. Integration of the previously reported SSRs with the high density RADseq markers joined two of the linkage groups reported in Yang et al., 2012 and 136 SSR markers are common between the two ‘AL1’ genetic maps. LG1-M and LG4-M from the SSR based map were integrated into LG1 in our RADseq based American lotus map. Most markers are collinear between the maps, which enables us to identify corresponding LGs and detect saturated regions. For instance, there are 15 markers (13 SSR markers and 2 SCAR markers) in the SSR based LG5-M, of which 13 SSR markers are shared in the RADseq map. Marker order between LG5-M and the corresponding RADseq LG3 is conserved except for SSR079e-s2 and SSR079d-s2 (Figure 3). Marker density is significantly higher in RADseq LG3 with an added 101 RADseq markers, increasing the map resolution.

**Figure 4:**
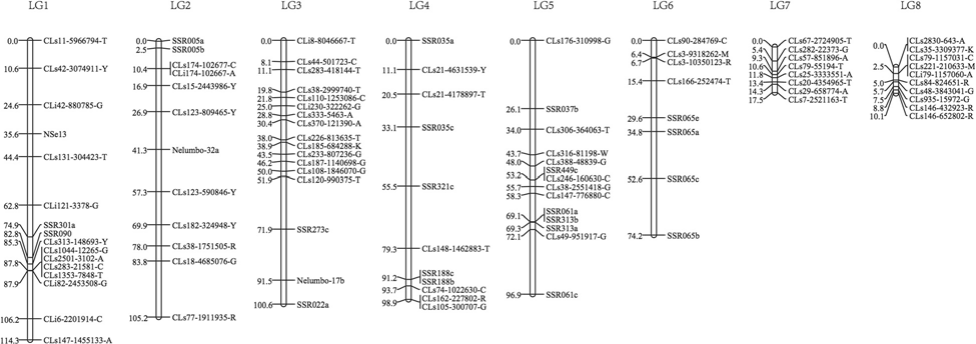
**Maternal linkage maps**.

**Figure 5:**
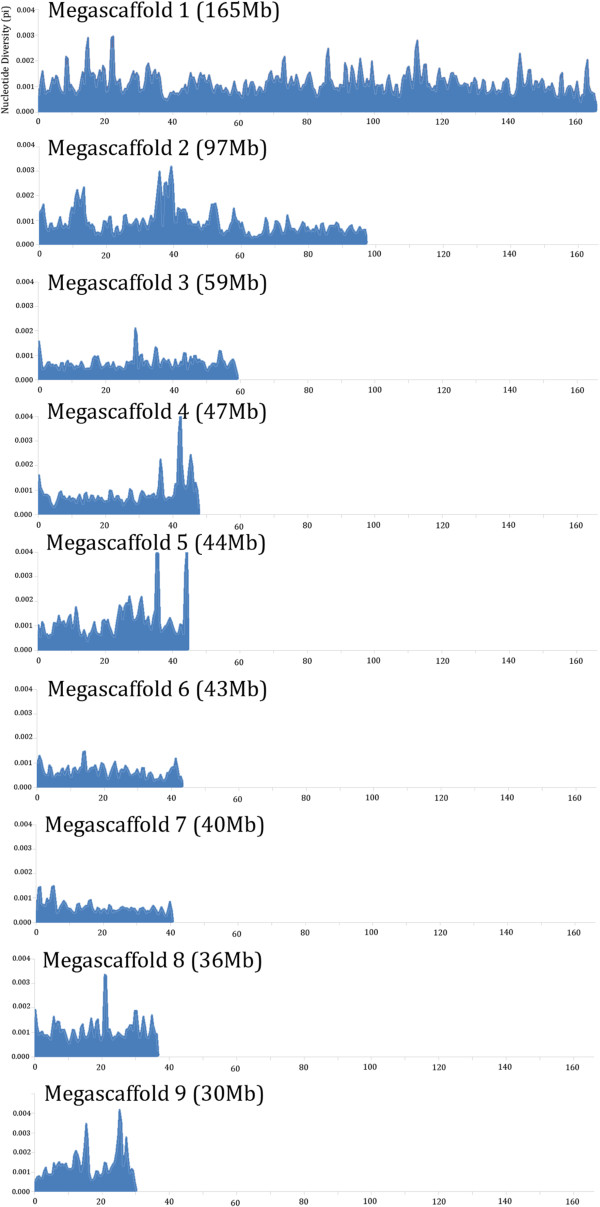
**Within genome heterozygosity of the Chinese lotus genome**. Nucleotide diversity (pi) is plotted along a sliding window of 500 Kb with 250 kb overlap for each of the 9 largest scaffolds.

Very few heterozygous loci in ‘China Antique’ were detected in RADseq tags. The dataset consisting of 105 SSR markers and only 147 RADseq markers was used to construct the female Chinese lotus genetic map. Ninety six markers (23 SSR markers and 73 RADseq markers) were mapped into 8 linkage groups (Figure 4). The total length of the Chinese lotus genetic map is 656.9 cM, with an average distance of 6.6 cM between adjacent markers, much higher than the American lotus map. The Chinese lotus linkage map is an improvement of the SSR based map. For instance, the SSR based LG5-F included only 3 SSR markers, where the corresponding RADseq based LG3 extended to 100.6 cM with 17 RADseq markers. The low marker density in the Chinese lotus genetic map is due to unexpectedly low level of heterozygosity within the genome of ‘Chinese Antique’. Heterozygosity was estimated by aligning the whole genome shotgun reads from the lotus genome sequencing project to the 9 megascaffold sequences. The genome has an average density of 0.3 SNP/InDels per kb (0.03%) with a non-uniform SNP distribution (Table 3, Figure 5). This residual heterozygosity of American lotus genome is 0.37%, 12 times that of American lotus [26].

**Table 3:** **Estimation of within genome heterozygosity in lotus**

Megascaffold	Within genome heterozygosity (variants/kb)
1	0.39
2	0.31
3	0.24
4	0.31
5	0.42
6	0.24
7	0.22
8	0.37
9	0.46
**Average**	**0.33**

### Application in genome assembly

The genome of the lotus variety ‘China Antique’ was sequenced with 94.2 Gb (101×) Illumina and 4.8 Gb (5.2×) 454 sequences, and the final assembly spans 804 Mb, 87% of the estimated 929 Mb lotus genome [26]. The well assembled lotus genome is largely due to the unexpected homozygosity of the ‘China Antique’ variety. Because of an insufficient number of RADseq markers from ‘China Antique’, only the high-density American lotus genetic map was used to anchor scaffold sequences and produce a chromosome sized assembly. The 9 anchored scaffolds span 543.4 Mb, accounting for 68% of the assembled genome (Table 4). In total, 88% of the markers in the American lotus linkage map could be anchored to the genome sequences. The relationship of genetic map and physical map was 1.1 Mb/cM.

**Table 4:** **Summary of mapping statistics for aiding the Chinese lotus genome assembly**

Linkage group	Distance (cM)	Markers	Markers per cM	SSRs	RADseq Bin Markers	Scaffold	Physical size (Mb)	Mb/cM	Involved scaffolds
LG1	97.7	203	2.1	39	164	1	165.9	1.70	91
LG2	75.8	79	1	16	63	3	59.25	0.78	17
LG3	69.1	114	1.6	14	100	2	97.35	1.41	37
LG4	58.3	83	1.4	14	69	7	40.61	0.70	17
LG5	51.1	61	1.2	18	43	6	47.89	0.94	4
LG6	48.2	80	1.7	19	61	8	36.68	0.76	17
LG7	44.9	33	0.7	3	30	9	30.01	0.67	21
LG8	27.7	32	1.2	10	22	5	44.88	1.62	17
LG9	21.5	13	0.6	3	10	10	20.78	0.97	13
Total	494.3	698	1.41	136	562		543.35	1.10	234

The 43 Mb megascaffold 4 anchored merely 5 markers in three bins compared to several hundred markers in each of the other scaffolds. Despite the few mapped markers, megascaffold 4 has a relatively normal polymorphism density comparable to the rest of the genome (Table 3, Figure 5), ruling out homology by decent as the cause. The low marker density of megascaffold 4 must be attributed to other factors.

## Discussion

The unmasked genome provided the largest number of markers but the lowest quality map, with over 15% of the markers anchored to incorrect scaffolds. These erroneous markers are likely repetitive sequences with multiple matches in the genome. Using gene models as a reference produced less than 10% markers compared to those using unmasked genome as a reference (776 vs. 8,501), and it produced two broken linkage groups and 12% erroneous markers. This low density map is missing most of the informative RADseq markers has little utility for marker assisted breeding or QTL analysis. The repeat masked genome provided the highest quality map with only 2% markers anchored to different scaffolds. It is unclear whether these 2% of markers are missing calls from linkage mapping, errors in genome assembly, or more likely a combination of both. Together this suggests most informative RADseq markers are located in single copy non-coding regions or within introns with relatively few found in the coding regions. A significant number of markers are also found in repetitive regions, but inclusion of these markers leads to incorrect genotype calling and subsequent errors in mapping.

The assembled draft genome of sacred lotus consists of 804 Mb with 57% repetitive sequences, and the 26,685 gene models accounted about 40 Mb, about 5% of the unmasked genome [26]. However, the scored RADseq markers using gene models as references are close to 9% the markers using unmasked genome as a reference, roughly twice as many as expected since gene models account for 5% of the assembled genome. The polymorphism rate in the genic regions should be lower than that of non-coding and repetitive sequences. The higher percentage of scored markers from the coding sequence is likely the result of misalignment of repetitive sequences causing incorrect scoring and skewed distribution. The repeat masked genome account for 43% of the assembled genome, but the scored markers are 48% those scored from unmasked genome, for the same reason. Our conclusion is that the optimal reference sequences for scoring RADseq markers for linkage mapping is the repeat masked draft genome, not the unmasked genome, and not the gene models.

Alignment against the unmasked genome produced significantly more markers (8,501) but only 65% mapped into LGs and a large number were unlinked or erroneous. Alignment against the gene models produced comparatively fewer markers (776) with 75% markers mapped, likely due to lower marker density. The repeat masked genome yielded the highest percentage of mapped markers, at 88%, strengthen the notion that the repeat masked genome is the best reference for calling RADseq markers. Using the unmasked genome as a reference is problematic because mis-scoring could occur in heterochromatic regions with abundant repetitive sequences, leading to subsequently errors in mapping. Using gene models as references would eliminate all markers in non-genic regions, which account for a vast majority of the 804 Mb assembled genome, resulting in an insufficient number of markers for constructing a saturated genetic map and for assisting genome assembly.

The relatively low marker density observed in ‘Chinese Antique’ is likely a consequence of its unexpectedly high level genome homozygosity. The estimated heterozygosity of ‘China Antique’ is 0.03%, much lower than the heterozygosity of American lotus ‘AL1’ which is twelve fold higher at 0.37%. Although lotus is an out-crossing plant, its cultivation and vegetative propagation via rhizomes over the past 7,000 years may have imposed a narrow genetic background [26]. The exceptional seed longevity observed in lotus might have further reduced the number of generations in its evolutionary history in addition to vegetative propagation. On the other hand, regions of low diversity may be explained by the smaller effective population size [27]. Population sizes are usually too small to detect variation between two loci. All possible genotypes for two loci must appear in the population in sufficient frequencies to allow statistical analysis [28]. The relatively fewer markers detected in Chinese lotus were possibly attributed to a smaller population size, though the relatively high-resolution genetic maps were constructed.

Though 3,894 RADseq markers and 136 SSR markers were mapped in the American lotus genetic map, the 9 linkage groups didn’t match the 8 chromosomes of the haploid genome, indicating that two LGs in the ‘AL1’ map come from one chromosome, but remain divided because of a large interval between the two linkage groups. Compared to the previous lotus genetic maps, the American lotus map presented here has a longer map distance, and higher marker density, with around 20 folds more markers. Two linkage groups (LG1-M and LG4-M) in the previously reported SSR map [7] were integrated into one linkage group (LG1) in America lotus maps because of the increasing marker number. The Chinese lotus genetic maps have an additional LG not found in the SSR maps, therefore the number of female genetic maps was consistent with that of the haploid chromosomes. The RADseq genetic maps improved the saturation and resolution compare to the previous lotus genetic maps, and provide a valid tool for genome research and crop improvement.

The American lotus genetic map was used to anchor the scaffold sequences of the lotus draft genome. The draft assembly spans 804 Mb, 87% of the total estimated genome size of 929 Mb [29], with a scaffold N50 of 4.3 Mbp before marker anchoring. 3,603 RADseq markers anchored 234 individual scaffold sequences into 9 megascaffolds with sizes roughly proportional to the lotus karyotype [26]. Furthermore, the high density markers and large scaffolds allowed for the orientation of most scaffolds in the genetic map. Integration of the genetic map and scaffold sequences resulted in a physical distance of 1.1 Mb/cM.

The high density lotus genetic map provides a framework for marker assisted breeding and QTL analysis. Several excellent traits exist in the two parents, for example plant size, leaf shape, petal shape and color [30]. Thus, a given trait might be improved by selection of markers which are linked to elite loci or alleles after QTL detection. Nuciferine, the main bioactive constituent of alkaloids in lotus leaves, is a key lipid-lowering substance. Recently, we detected significant variation in nuciferine content between *Nelumbo nucifera* ‘China Antique’ (299.0 μg/g FW) and *N. lutea* ‘AL1’ (1410.1 μg/g FW). Mapping quantitative trait loci of nuciferine content based on high-density lotus genetic map is essential to guide molecular breeding of lotus cultivars with high nuciferine content. Moreover, several excellent traits might be combined in one lotus plant, thereby producing a new cultivar, through a series of crosses and marker-assisted selection (MAS). In addition, the RADseq markers could be used as shared anchors to compare genetic and physical maps. The high density genetic maps presented here can facilitate comparative genomic analysis, genome assembly, QTLs analysis and molecular assisted breeding.

## Conclusions

In this work, we identify the optimal reference sequence for scoring RADseq markers using the unmasked scaffolds, repeat masked scaffolds, and gene models as references. The repeat masked genome provided the highest quality map as a reference with the highest accuracy rate. The unmasked genome provided the largest number of markers but the lowest quality map, which is likely due to repetitive sequences with multiple matches in the genome. Using gene models as a reference produced the least markers compared to those of unmasked genome and repeat masked sequences, and generated low density map because of missing most of the informative RADseq markers.

The high density genetic map of American lotus was constructed with 562 bins (3,894 RADseq markers) integrating with 136 SSR markers. The total length of the American lotus genetic map is 494.3 cM, with an average distance of 0.7 cM between adjacent markers/bins. The relatively low marker density observed in ‘Chinese Antique’ is likely a consequence of its unexpectedly high level genome homozygosity. The high density genetic maps significantly improved the saturation and resolution compare to the previous lotus genetic maps, and facilitated genome research and crop improvement.

## Methods

### Plant material

A mapping population of lotus was generated by crossing *Nelumbo nucifera* ‘Chinese Antique’ (female) and *N. lutea* ‘AL1’ (male), yielding 51 F_1_ individuals. The parents and F_1_ individuals from this cross were maintained at Wuhan Botanical Garden of the Chinese Academy of Sciences. DNA was extracted from young leaves from 51 seedlings and the two parents using a modified cetyltrimethyl ammonium bromide (CTAB) method described by Doyle [31]. After quality assessment, DNA concentrations were adjusted to 100 ng/μL for RADseq library preparation.

### RADseq library preparation

A modified double digest Restriction Associated DNA (ddRAD) procedure [32] was used for RADseq library preparation. ddRADseq eliminates the random shearing and end repair steps used in traditional RADseq library preparation, instead relying on a double restriction enzyme digestion, which leads to at least five-fold cost reduction in library construction [32]. Briefly, genomic DNA (1 μg) from each F_1_ sample was digested with two restriction endonucleases, *Nsi*I and *Mse*I (NEB, USA) which recognize a 6-nucleotide sequence (5′ATGCA/T3′) and a 4- nucleotide sequence (5′T/TAA3′), respectively. After digestion at 37°C for 2 hours in a 50 μl reaction, each sample was heat-inactivated at 80°C for 20 min. The ligation reaction was performed with 8 μl of 0.1 μM modified Solexa P1 Adaptor and 1 μl of 10 μM Solexa P2 Adaptor (Illumina, USA), along with 30 μl of the digested DNA sample, 5 μl of 10 mM rATP (Promega, USA), 10×NEB Buffer 3, and 400 U T4 DNA ligase (NEB, USA), 2.75 μl H2O at room temperature for 4 hours. P1 and P2 Adaptor sequences are as follows: P1 top: 5′-GATCTACACTCTTTCCCTACACGACGCTCTTCCGATCTxxxxxTGCA-3′ (xxxx indicates barcode), P1 bottom: 5′yyyyAGATCGGAAGAGCGTCGTGTAGGGAAAGAGTGTAGATC-3′ (yyyy indicates reverse complement of barcode); P2 top: 5′-TAGATCGGAAGAGCACACGTCTGAACTCCAGTCACCTTGTAATCAGAACAA-3′, P2 bottom: 5′-CAAGCAGAAGACGGCATACGAGATTACAAGGTGACTGGAGTTCAGACGTGTGCTCTTCCGATC-3′.The sample amounts for two parents were two times more than those for each F_1_ sample. Samples from each individual were heat-inactivated at 65°C for 20 min and pooled together. Pooled DNA sample was run out on a 1% agarose (Sigma, USA), 0.5 × TBE gel, and DNA fragments 200 bp to 500 bp was isolated using a Min Elute Gel Extraction Kit (Qiagen, Germany). Purified product was amplified with 25 uL Phusion Master Mix (NEB, USA), 2 uL of 10 uM modified Solexa amplification primer mix (Illumina, USA), and H2O to 50 ul. Phusion PCR proceeded following product guidelines (NEB, USA) for 18 cycles. Samples were purified again using the QIA quick PCR purification kit (Qiagen, Germany), and diluted to 10 nM for Illumina HiSeq2000 sequencing.

### SNP discovery and genotyping with three different references

Low-quality reads (Q score <20) and reads lacking a correct barcode were filtered out. SNP/InDel calling was processed using a custom protocol, custom Shell and Perl scripts. Briefly, 100 bp raw sequence reads were sorted by barcode using the Stacks package [33], and trimmed to 92 nucleotides to remove flanking barcode sequences. Identical RADseq tags and those with one or two mismatches (SNPs or 1–2 bp InDels), were aligned using Novoalign software (http://www.novocraft.com/), and the SAM output file was converted to BAM format and sorted using the SAM tools suite [34].

For estimating the optimal reference, RADseq reads from the parents were aligned to the scaffold sequences, gene models, and repeat masked scaffold sequences from the Sacred lotus draft genome [26], respectively. To further minimize errors in assigning markers, clusters with less than 4 or more than 200 reads were discarded. In a suitably robust cluster, mismatches among different individuals were considered putative polymorphisms. Based on different reference sequences, three sets of polymorphic marker between ‘Chinese Antique’ and ‘AL1’ were identified respectively by pairwise grouping from each genotype. To score F1 population genotypes, progeny sequencing tags were also aligned into clusters, and compared to the parental marker panel to identify genotypes.

Neighboring markers were binned based on similarity scores and linked markers within each bin were counted using custom Perl scripts. The similarity scores were derived from a pairwise array, which calculated the sum of identical and missing genotype scores across all progeny.

### SSR markers for mapping

The primers prefixed by ‘Nelumbo’, ‘NSh’, ‘NNEST’ and ‘NSe’ were derived from genomic or EST sequences of *N. nucifera*[35–38]. The markers prefixed by ‘SSR’ were developed in an ‘AL1’ × ‘China Antique’ F_1_ population [7]. All PCR amplifications proceeded in a 10 μL reaction mixture which containing 50 ng of DNA, 0.8 mmol L^−1^of each primer, 2.0 mmol L^−1^ Mg^2+^, 0.2 mmol L^−1^dNTPs, 10× PCR buffer, and 0.5 U Taq DNA polymerase. PCR amplification was carried out with initial denaturation at 94°C for 5 min; 40 s at 94°C, 30 s at the appropriate annealing temperature, and 40 s of extension at 72°C for 35 cycles with a final elongation at 72°C for 10 min.

### Construction and comparison of genetic linkage maps

Three sets of polymorphic markers refereed from scaffold sequences, gene models, and repeat masked scaffold sequences respectively, were used for constructing genetic linkage maps with JoinMap 4.1 [39]. Genotypes were converted into “CP” (cross pollinator) population codes based on the genotypes of parents. Markers displaying a segregation ratio greater than 3:1 for markers with an expected ratio of 1:1 and greater than 10:1 for markers with an expected ratio of 3:1 were excluded from map construction. Grouping was carried out using a regression mapping algorithm and a maximum recombination frequency of 0.40. Kosambi’s function was applied to compute map distance (cM). The high quality RADseq markers combined with SSR markers were assigned to construct ultimate genetic maps.

### Estimation of within genome heterozygosity

Over 600 million Illumina reads (representing over 60× coverage) from the lotus genome sequencing project [26] were aligned to the 9 assembled megascaffolds to estimate within genome heterozygosity. Reads were aligned using Novoalign software (http://www.novocraft.com/) under default parameters, and the SAM output file was converted to BAM format and sorted using the SAM tools suite [34]. SNPs and InDels were called in SAM tools with a minimum depth of 10× and maximum read depth of 120× to avoid errors due to low coverage and repetitive regions.

### Availability of supporting data

The restriction site associated DNA sequencing (RADseq) reads were deposited at NCBI with accession number:PRJNA243020. (http://www.ncbi.nlm.nih.gov/bioproject/243020).

## Abbreviations

SNP: Single-nucleotide polymorphism
ddRAD: Double digest restriction associated DNA.
